# Heterogeneous vancomycin-intermediate susceptibility in a community-associated methicillin-resistant *Staphylococcus aureus *epidemic clone, in a case of Infective Endocarditis in Argentina

**DOI:** 10.1186/1476-0711-10-15

**Published:** 2011-04-28

**Authors:** Claudia Sola, Ricardo O Lamberghini, Marcos Ciarlantini, Ana L Egea, Patricia Gonzalez, Elda G Diaz, Vanina Huerta, Jose Gonzalez, Alejandra Corso, Mario Vilaro, Juan P Petiti, Alicia Torres, Ana Vindel, Jose L Bocco

**Affiliations:** 1 Centro de Investigaciones en Bioquímica Clínica e Inmunología (CIBICI-CONICET); Departamento de Bioquímica Clínica; Facultad de Ciencias Químicas; Universidad Nacional de Córdoba; Córdoba, Argentina; 2 Servicio de Infectología y Microbiología del Hospital Militar de Córdoba, Córdoba, Argentina; 3 Laboratorio de Microbiología SUOEM, Córdoba, Argentina; 4 Instituto Nacional de Enfermedades Infecciosas Dr. Carlos G. Malbrán, Buenos Aires, Argentina; 5 Laboratorio de Microbiología Hospital Privado Centro Médico de Córdoba, Córdoba, Argentina; 6 Centro de Microscopía Electrónica, Facultad de Ciencias Médicas, Universidad Nacional de Córdoba, Córdoba, Argentina; 7 Laboratorio de Infecciones Nosocomiales, Instituto de Salud Carlos III, Centro Nacional de Microbiología, 28220 Majadahonda, Madrid, Spain

## Abstract

**Background:**

Community-Associated Methicillin Resistant *Staphylococcus aureus *(CA-MRSA) has traditionally been related to skin and soft tissue infections in healthy young patients. However, it has now emerged as responsible for severe infections worldwide, for which vancomycin is one of the mainstays of treatment. Infective endocarditis (IE) due to CA-MRSA with heterogeneous vancomycin-intermediate susceptibility-(h-VISA) has been recently reported, associated to an epidemic USA 300 CA-MRSA clone.

**Case Presentation:**

We describe the occurrence of h-VISA phenotype in a case of IE caused by a strain belonging to an epidemic CA-MRSA clone, distinct from USA300, for the first time in Argentina. The isolate h-VISA (SaB2) was recovered from a patient with persistent bacteraemia after a 7-day therapy with vancomycin, which evolved to fatal case of IE complicated with brain abscesses. The initial isolate-(SaB1) was fully vancomycin susceptible (VSSA). Although MRSA SaB2 was vancomycin susceptible (≤2 μg/ml) by MIC (agar and broth dilution, E-test and VITEK 2), a slight increase of MIC values between SaB1 and SaB2 isolates was detected by the four MIC methods, particularly for teicoplanin. Moreover, Sab2 was classified as h-VISA by three different screening methods [MHA5T-screening agar, Macromethod-E-test-(MET) and by GRD E-test] and confirmed by population analysis profile-(PAP). In addition, a significant increase in cell-wall thickness was revealed for SaB2 by electron microscopy. Molecular typing showed that both strains, SaB1 and SaB2, belonged to ST5 lineage, carried *SCCmec*IV, lacked Panton-Valentine leukocidin-(PVL) genes and had indistinguishable PFGE patterns (subtype I2), thereby confirming their isogenic nature. In addition, they were clonally related to the epidemic CA-MRSA clone (pulsotype I) detected in our country.

**Conclusions:**

This report demonstrates the ability of this epidemic CA-MRSA clone, disseminated in some regions of Argentina, to produce severe and rapidly fatal infections such as IE, in addition to its ability to acquire low-level vancomycin resistance; for these reasons, it constitutes a new challenge for the Healthcare System of this country.

## Background

Community-associated methicillin resistant *Staphylococcus aureus-*(CA-MRSA) has rapidly become the main cause of *S. aureus *infections worldwide since 1993. These non-multiresistant strains frequently harbor staphylococcal-cassette-chromosome-(SCC*mec*) types IV or V and Panton-Valentine leukocidin-(PVL) genes. The worldwide increase of CA-MRSA infections is due to the dissemination of some epidemic CA-MRSA clones harboring *pvl *genes (PVL^+^), with a specific geographical pattern, these are frequently designated by their multilocus sequence type (MLST) or by their pulsed field gel electrophoresis (PFGE) pattern **(**ST1, ST8-USA300, ST30, ST59, ST93 and ST80) [1-4].

Although CA-MRSA was initially related to skin and soft tissue infections, it has now emerged as a cause of severe infections worldwide [1]. Vancomycin has traditionally been the mainstay of therapy for these serious MRSA infections, thereby the emergence of resistance to this agent is of great concern [5,6]. Heterogeneous vancomycin intermediate *S. aureus *(h-VISA) is an isolate of *S. aureus *with a vancomycin MIC within the susceptible range (≤2 μg/ml) when tested by routine methods, containing subpopulations of cells (typically at a frequency of ≤10^-5 ^to 10^-6^) with intermediate resistance to vancomycin (VISA: MIC 4-8 μg/ml) [5]. This type of resistance is associated with a thickened cell-wall that prevents vancomycin action over its target within the cytoplasmic membrane. The exact mechanisms leading to this thickening have not been determined yet [5].

Lately, cases of infective endocarditis (IE) caused by CA-MRSA, mainly associated with the USA300 genotype have been reported [7-9]. More recently, phenotype h-VISA related to USA300 CA-MRSA clone has also been described [3,10-12]. On the other hand, **t**he emergence of a ST5-IV clone PVL^+ ^among CA-MRSA strains in Argentina was detected in 2005 [4,13].

We report the occurrence of h-VISA phenotype in a case of IE caused by this epidemic CA-MRSA clone for the first time in this country.

## Case Presentation

**On April 4, 2009**, a 73-year-old female with sudden weakness in the extremities was admitted at Institution A. On admission, she was afebrile and the head computed tomography (CT) was normal. She had underlying chronic rheumatic valve disease and chronic atrial fibrillation. She had no previous exposure to healthcare personnel or hospital environment during the previous year. Forty-eight hours after admission, the patient developed fever (38°C) and depression of the sensorium; hence, she was transferred to the Intensive Care Unit (ICU). Abdominal CT scan and echo-Doppler of the carotid vessels were normal.

**Figure 1:**
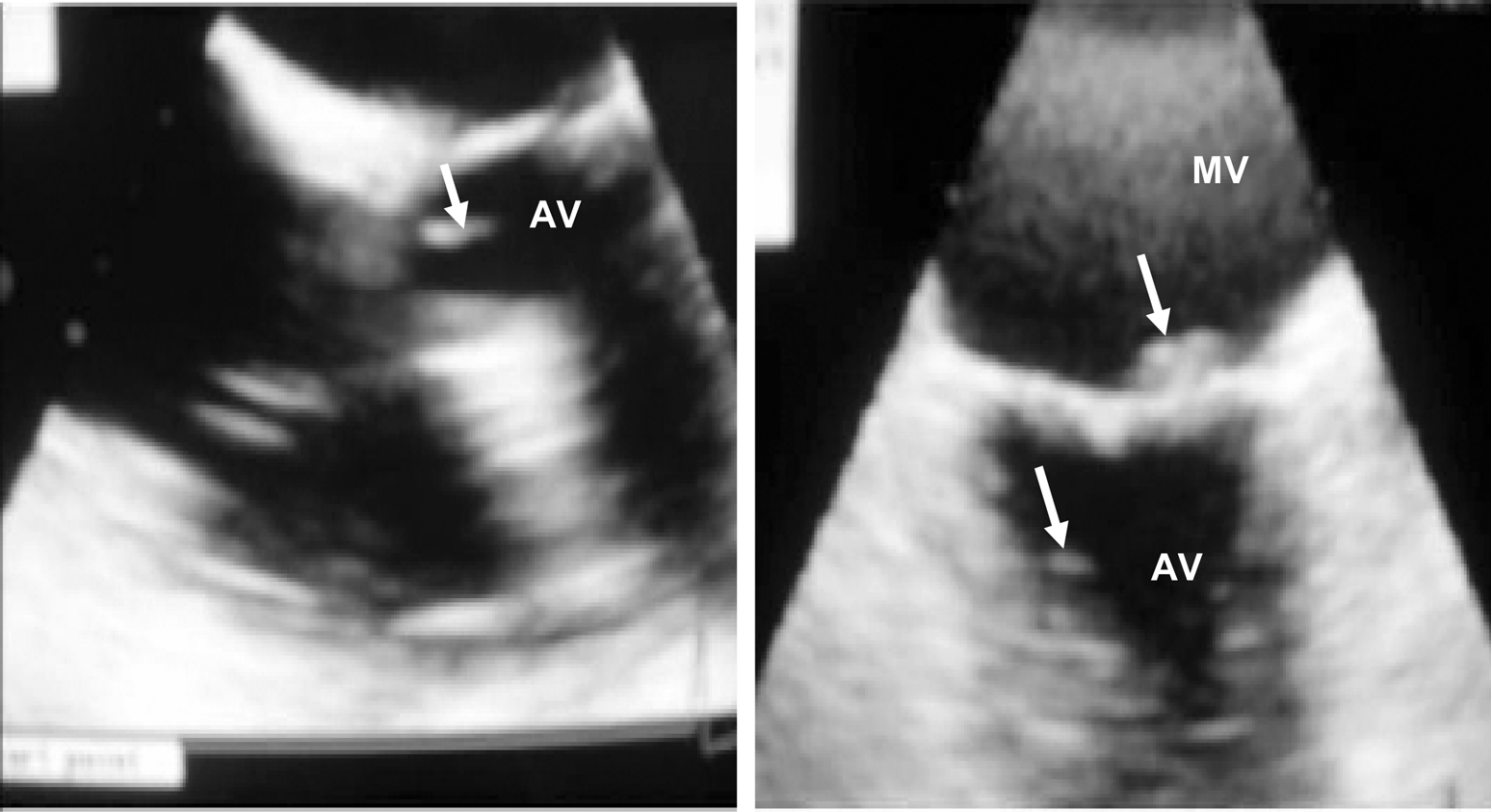
**Transesophageal echocardiography of a patient with infective endocarditis caused by CA-MRSA with phenotype h-VISA, Argentina**. **Left**: On April 12, the highly mobile vegetation on the aortic valve (AV) of 20 × 41 mm (white arrow). **Right**: On April 24, slight decrease in the aortic valve (AV) vegetation (white arrow) and a new vegetation on the mitral valve (MV) (10 × 4.6 mm) of low motility (white arrow).

MRSA grew in three blood cultures (SaB1-strain) and one urine culture (SaU-strain) taken upon admission in the ICU on April 7. A transthoracic echocardiography (TE) showed a filamentous mobile image attached to the ventricular side of the aortic valve, which resembled vegetations, suggesting a MRSA infection that met Duke's criteria for IE [14]. These isolates were susceptible to ciprofloxacin, gentamicin, rifampicin, tetracycline, trimethoprim-sulfamethoxazole-(TMP-SMX) and linezolid by disk diffusion and broth microdilution methods (see Additional file 1, Table S1) [15] and resistant to erythromycin and clindamycin (inducible resistance, D-test positive) [15]. In addition, vancomycin MIC was 1 μg/ml by broth dilution method [15]. Vancomycin treatment (15 mg/kg every 12 h) was initiated on April 7 and the patient was transferred to "Hospital-Militar-Regional-Córdoba-(HMRC)" for further evaluation on April 8. Antibiotic levels in blood were not available, so the same dose of vancomycin was continued; which is recommended to maintain the level of vancomycin in the valley of 10-15 μg/ml. The patient had fever of 39.2°C accompanied by Janeway's lesions on the hands. On April 9, valve replacement was postponed because of a worsening of the neurological symptoms and hypotension. Roth's spots were detected. The brain CT revealed no changes. On April 12, the patient presented hemodynamic and neurological improvement; a transoesophageal echocardiography (TEE) showed a highly mobile vegetation on the aortic valve (Figure 1). On April 14, the patient had altered mental status and persistent fever. Brain magnetic resonance-image-MRI with gadolinium showed multiple abscesses (Additional file 2, Figure S1). On April 15, since the patient worsened, valve replacement was postponed again; treatment failure was suspected and trimethoprim/sulfamethoxazole (15 mg/kg/day) was added to vancomycin. On April 17, the patient remained febrile, three new samples for blood culture taken on April 14 revealed MRSA (strain SaB2) with the same susceptibility to antibiotics, except for vancomycin, which reached a MIC of 2 μg/ml (broth dilution [15]. On April 20, the patient was afebrile. Three additional sets of blood cultures were drawn providing negative results. On April 24, a control TEE (Figure 1) showed a slight decrease of the size of the aortic valve vegetation and a new vegetation on the mitral valve. The patient had evident deterioration of mental and physical status and died on April 25 before surgical intervention.

The emergence of h-VISA phenotype in a CA-MRSA was suspected based on the rapid and fatal outcome of the patient, the facts that the IE was caused by MRSA without multiresistance to antibiotics, failure of vancomycin therapy (defined as persistent fever and bacteraemia longer than 7 days after onset of therapy) and increase of vancomycin MICs values (1 to 2 μg/ml) within therapy. This prompted us to further analyze these strains. The blood (SaB1 and SaB2) and urine (SaU) MRSA isolates were sent to the CIBICI ("Centro de Investigaciones en Bioquimica Clinica e Inmunologia"; UNC-CONICET); for additional analysis including molecular and genetic characterization.

### Phenotypic and genotypic characterizations of MRSA

**Figure 2:**
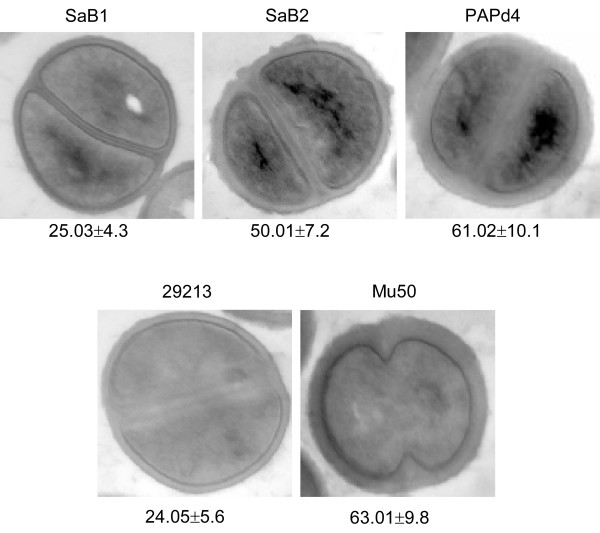
**Transmission electron microscopy of initial isolate (SaB1)-VSSA vs. after persistent bacteremia isolate (SaB2)-h-VISA**. Comparison of the cell thickness among the pair of strains (SaB2 vs SaB1) and one derivative VISA (CIM 4 μg/ml) obtained on PAP (PAPd4). Quality control strains included *S. aureus *ATCC 29213 and Mu50, control, negative and positive for VISA, respectively. Magnification: × 60,000. Values given under each image are mean ± SD of the cell wall thickness in nanometers. Student's t test demonstrated that this augment in cell wall thickness was statistically significant (*p <*0.001).

The slight increase of MIC values between the initial (SaU and SaB1) and subsequent (SaB2) isolates was detected by four MICs methods [15], particularly for teicoplanin (Table 1). The isolates were also sent to the "Instituto-Nacional-de-Enfermedades-Infecciosas Dr. Carlos G. Malbrán" to independently confirm the MIC results for vancomycin and teicoplanin. Moreover, SaB2 was classified as h-VISA by three screening methods, MHA5T-screening agar, Macromethod-E-test-(MET) and GRD E-test (Table 1) [5]. Although MRSA SaB2 was vancomycin susceptible by MIC, it grew in BHI agar containing 4.0 μg/mL of vancomycin at a frequency of 1.6 × 10^-6 ^on population analysis profile-(PAP) [16,17], which is consistent with h-VISA (Additional file 3, Figure S2). Vancomycin MICs by broth-dilution; agar-dilution and E-test were determined for these derivatives and all were between 4 and 8 μg/ml. Neither MRSA SaB1 isolate nor *S. aureus *ATCC 29213 grew in BHI agar with 4 μg/ml. In addition, electron microscopy examination [5,18] of SaB2 and one derivative VISA obtained on PAP, revealed that the cell-wall of both isolates was thicker (*p <*0.001) than the parent strain SaB1 (Figure 2), a feature observed for most strains with VISA and h-VISA phenotypes. Moreover, the h-VISA phenotype found in strain SaB2 was unstable, since vancomycin MICs in all isolates with MICs ≥2 μg/ml reverted to MICs ≤1 μg/ml (susceptible phenotype-VSSA) after multiple subcultures in absence of vancomycin. Importantly, the detection by PCR [11] of *vanA and vanB genes *was negative.

**Table 1:** Susceptibility of urine-(SaU) isolate and blood isolates obtained before (SaB1) and during (SaB2) vancomycin therapy

Isolates	Broth dilution μg/ml		Agar dilution μg/ml		*E-test* μg/ml		VITEK 2 μg/ml		MET μg/ml		GRD *strip* μg/ml		MHA5T
	**VAN**	**TEIC**	**VAN**	**TEIC**	**VAN**	**TEIC**	**VAN**	**TEIC**	**VAN**	**TEIC**	**VAN** **24 h/48 h**	**TEIC** **24 h/48 h**	**TEIC**

SaU	1	1	1	1	1	1	1	≤ 05	4	8	1/1	2/2	-
SaB1	1 (1)*	1	1	1	1 (1.5)*	1 (1)*	1	≤ 05	4	8	1/1	2/2	-
**SaB2**	2 (2)*	**4**	**2**	**4**	2 (2)*	2 (1.5)*	**2**	**4**	**12**	**12**	**4/4**	**12/16**	+

**MIC: **Minimum inhibitory concentration, **VAN**: vancomycin, **TEIC**: teicoplanin, **MET**: **Macromethod E-test**, 200 μl of inoculum at 2 McFarland standard suspensions onto BHI Agar, positive result: VAN MIC of ≥8 and TEIC MIC of ≥8 μg/ml or TEIC MIC of ≥12 μg/ml; **GRD strip**, Etest Glycopeptide-resistance (AB bioMérieux) detection strip were used as described by the manufacture:**GRD positive (GISA o h-GISA): VAN o TEIC ≥ 8** μg/ml; **MHA5T screening**, Mueller Hinton Agar with 5 μg/ml of TEIC, 10 μl of inoculum at 0.5 McFarland-standard direct-colony suspensions. Any growth was considered positive. All screening methods were read at 24 and 48 h and incubated at 35°C. (MIC)*: MIC results from the Instituto Nacional de Enfermedades Infecciosas Dr. Carlos G. Malbran. The strains h-VISA-Mu3 and VISA-Mu50 and the vancomycin-susceptible S. aureus strain ATCC 29213 were tested for all methods in parallel, as positive and negative controls, respectively.

The clinical isolates (SaU, SaB1 and SaB2) and the derivatives obtained on PAP were analyzed by PFGE, MLST, SCC*mec *and *spa *typing, in addition to the detection of virulence genes by PCR, as previously reported [4]. Molecular typing revealed that all these isolates had indistinguishable pulsotypes (subtype I2) (Additional file 4, Figure S3) and were characterized as ST5, *agr-*2, *spa*-t045, associated to SCC*mec*-IV. The *egc (seg, sei, sem, sen and seo) *and *lukED *genes were detected among all the virulence genes analyzed by PCR in these three isolates. They do not harbor *pvl and sea *genes. The results confirmed the isogenic nature of these isolates and their clonality with the CA-MRSA clone (pulsotype I) detected in our country (Additional file 4, Figure S3).

## Discussion

Currently, PVL negative CA-MRSA strains belonging to some successful PVL positive CA-MRSA clones (ST1, ST59, ST5) or to the new one (ST72) have been detected, particularly in Europe and Asian-Pacific countries [1,19-21]. In the case of the CA-MRSA IE reported here, the molecular typing revealed that although these strains share the same successful lineage ST5 with CA-MRSA (ST5-IV) and -HA-MRSA (ST5-I) epidemic clones disseminated in this country, their PFGE pattern is closer to that of the CA-MRSA clone (one band of difference with I1, additional Figure 3). Moreover, this isolate carries the *SCCmec *type IV and is non-multiresistant to antibiotics. Consequently, results identify a PVL-negative variant (PVL^-^) of the ST5-IV CA-MRSA epidemic clone detected in Argentina. Importantly, this clone belongs to lineage ST5, which has been demonstrated [5] to develop the VISA phenotype, but so far associated to HA-MRSA clones (ST5-II), unlike our case. These characteristics could confer another selective advantage for its dissemination in Argentina and neighboring countries.

Presently, strains of MRSA demonstrating the h-VISA or VISA phenotype have been reported in many countries worldwide, but in Latin America, cases with these phenotypes have been reported only in Northern countries and Brazil [3,5]. Furthermore, only one case of CA-MRSA infective endocarditis from Brazil has been reported for South America, but not associated to a h-VISA strain like our case[22]. In Argentina, the h-VISA phenotype in MRSA strains was detected for the first time in 2009. Thus, our case is the first one associated to persistent bacteremia under vancomycin therapy. Three additional cases were independently detected in Buenos Aires during the same year (presented at "Congreso Argentino de Infectología -SADI 2010" and at "XX-Congreso Latinoamericano de Microbiología; Montevideo-2010", unpublished data). Even in infections with "high-risk" of presence of h-VISA phenotype, such as IE, the prevalence of h-VISA varies significantly and is potentially associated with non-clinical factors, such as geographic location [14]. Hence, the knowledge about general and molecular epidemiology of h-VISA phenotype in different countries is essential for effective prevention and therapy.

On the other hand, the cases of CA-MRSA IE subsequently published occurred in healthy young patients without classical risk factors for IE [7]. This report, along with another case of CA-MRSA IE recently described in Italy, [8] support the importance of considering this emergent pathogen as a potential cause of IE for subjects with risk for IE (elderly patients, pre-existing heart diseases, etc). Hence, our report adds new information about CA-MRSA associated to h-VISA phenotype epidemiology, which is a matter of great interest in clinical microbiology and molecular epidemiology worldwide.

In addition, our case highlights the difficulties of laboratory detection of h-VISA-phenotype, particularly when it is unstable and arises from a fully susceptible VSSA isolate (vancomycin MIC ≤1 μg/ml) under treatment with vancomycin. Furthermore, upon detection, the choice of appropriate therapy still remains difficult [5,6]. Daptomycin, vancomycin, teicoplanin, linezolid, TMP-SMX, and quinupristin-dalfopristin are potential options for the treatment of MRSA bacteraemia [6,23,24]. The treatment of our patient raises several questions and reflects the lack of evidence-based data to guide the choice of therapy for CA-MRSA IE caused for isolates expressing h-VISA phenotype with vancomycin MICs of 2 μg/ml [5,6]. Our patient received vancomycin as first line therapy at recommended doses [6,23]. However, due to the clinical worsening of the patient, which prevented the valvular surgery, in addition to the presence of brain abscesses, we decided to add TMP-SMX to vancomycin, even before knowing that vancomycin MIC value had risen to 2 μg/ml. TMP-SMX has bactericidal activity *in-vitro *and it may be considered to be an alternative therapy to vancomycin for MRSA infection [6,24-26]. In our patient, although the last set of blood cultures may have been negative for different reasons, including changes in renal function, TMP-SMX apparently contributed to bacteraemia clearance. Only few cases of patients with persistent bacteraemia have been treated with TMP-SMX in addition to vancomycin, late in the course of bacteriemia, but these are not enough to assess the clinical outcome [27]. Daptomycin was not considered due to the potential risks in patients with bacteremia, in which a deep focus of infection has not been surgically debrided, as well as for patients with left-side endocarditis [28], two characteristics seen in our patient.

One of the limitations of this report is the absence of measurements of vancomycin serum concentrations due to technical problems of the clinical laboratory at that time. Hence, although we think that, considering the clinical characteristics of the patient, the dose of vancomycin that she received should have been enough to reach or even exceed the recommended therapeutic level [6,27], we cannot demonstrate it. In summary, the failure of vancomycin to eradicate this CA-MRSA strain was likely due to the presence of several important factors: I) high-bacterial-load during infection, combined with the impossibility to perform the cardiac surgery, II) slow bactericidal mechanism of vancomycin, III) the ability of this CA-MRSA clone to develop h-VISA phenotype and IV) the virulence of the CA-MRSA strain, possibly related to the metastatic complications and rapid progression [5,23].

## Conclusions

This report demonstrates the ability of this epidemic CA-MRSA clone, disseminated in some regions of Argentina, to cause severe and rapidly fatal infections such as infective endocarditis, in addition to its capacity to acquire low-level vancomycin resistance; for this reason, it has become a challenge for the healthcare system in our country. Therefore, systematic surveillance along to molecular epidemiology and quick diffusion of the results in each region are crucial for proper management of these infections.

## Consent

Written informed consent was obtained from the patient's son for publication of this case report and any accompanying images. A copy of the written consent is available for review by the Editor-in-Chief of this journal

## Competing interests

The authors declare that they have no competing interests.

## Authors' contributions

CS participated in the design and coordination of the study, drafted the manuscript and carried out part of the molecular typing of the isolates. RL: participated in clinical infectious disease's diagnosis and treatment, helped drafting the manuscript and revised the manuscript critically for important intellectual content. MC and JG: contributed to clinical care of the patient and the collection and analyses of the clinical data. ALE and VH: carried out the methodology of glycopeptides susceptibility and part of the molecular typing of the isolates. PG, ED and MV: carried out the identification and antibiotic susceptibility of the isolates. AC: contributed to the methodology of glycopeptides susceptibility of the isolates. JPP and AT: carried out the transmission electron microscopy of the isolates.

AV: carried out part of the molecular typing of the isolates. JLB participated in the design of the study, helped drafting the manuscript and revised the manuscript critically for important intellectual content. All authors read and approved the final manuscript.

## Supplementary Material

Additional file 1 **Table S1: Antimicrobial susceptibility profile of urine-(SaU) isolate and blood isolates obtained before-(SaB1) and during-(SaB2) **vancomycin therapy**. CLSI: **Clinical and Laboratory Standards Institute, **MIC: **Minimum inhibitory concentration by broth microdilution (VITEK 2) per CLSI guideline, R*: Inducible Clindamycin Resistance. Click here for file

Additional file 2 **Figure S1: MRI (two slices) showing brain abscesses (white arrows) in a patient with CA-MRSA infective endocarditis**. MRI: magnetic resonance image (with gadolinium). Click here for file

Additional file 3 **Figure S2: Vancomycin population analysis profiles of initial isolate-(SaB1)-VSSA and after persistent bacteremia isolate-(SaB2)-h-VISA, despite vancomycin therapy SaB1: initial isolate, prior to vancomycin therapy**. Also, hVISA and VISA reference strain Mu3 and Mu50 respectively and VSSA strain ATCC 29213. Briefly, PAP was performed by serial dilution of overnight BHIB culture and inoculation of BHI agar containing 0 to 8 μg/ml of vancomycin. Colonies were counted after incubation for 48 h in air at 35°C and plotted on a graph of the number of CFU/ml versus vancomycin concentration. Click here for file

Additional file 4 **Figure S3: PFGE-Analysis confirmed the clonality of the clinical isolates (SaU-SaB1-SaB2), belonging to CA-MRSA clone ST5-IV-PVL+**. Sma I restriction patterns were indistinguishable for SaU (urine isolate), SaB1 (initial blood isolate) and SaB2 (later blood isolate), 1-5: five derivatives from SaB2 in PAPs (CIM ≥4 μg/ml), PFGE DNA pattern of representative of major clonal types belonging to ST5 lineage, both CA and HA-MRSA from Argentina, to be compared with subtype I2 CA-MRSA (ST5-IV-PVL¯): CA-MRSA I1 (ST5-IV-PVL^+^), HA-MRSA A1-Cordobes/Chilean clone (ST5-I) and HA-MRSA C1 Pediatric clone (ST100-IV). I6 refers to the PFGE pattern of a MSSA isolate from Córdoba, which shows a unique band difference with the I2 subtype, isolated from our patient. NC: NCTC 8325 control strain L: DNA molecular size markers in kb (lambda DNA ladder, Promega). Click here for file
