# Supplementary material for: Heterogeneous vancomycin-intermediate susceptibility in a community-associated methicillin-resistant Staphylococcus aureus epidemic clone, in a case of Infective Endocarditis in Argentina
Source: Ann Clin Microbiol Antimicrob. 2011 Apr 28;10:15. doi: 10.1186/1476-0711-10-15 (PMC3111347; doi:10.1186/1476-0711-10-15)
Supplement: Additional file 1 — Table S1: Antimicrobial susceptibility profile of urine-(SaU) isolate and blood isolates obtained before-(SaB1) and during-(SaB2) vancomycin therapy. CLSI: Clinical and Laboratory Standards Institute, MIC: Minimum inhibitory concentration by broth microdilution (VITEK 2) per CLSI guideline, R*: Inducible Clindamycin Resistance. [file 1476-0711-10-15-S1.DOC]

## Additional file 1, Table S1

|  | **MIC (µg/ml) and CLSI interpretation** | | |
| --- | --- | --- | --- |
| Antimicrobial drug | SaU | SaB1 | SaB2 |
| Oxacillin | ≥ 4 R | ≥ 4 R | ≥ 4 R |
| Clindamycin | ≤ 0.25 R* | ≤ 0.25 R* | ≤ 0.25 R* |
| Erythromycin | ≥ 8 R | ≥ 8 R | ≥ 8 R |
| Trimethoprim-Sulfamethoxazole | ≤ 10 S | ≤ 10 S | ≤ 10 S |
| Gentamicin | ≤ 0.5 S | ≤ 1 S | ≤ 1 S |
| Tetracycline | ≤ 1 S | ≤ 0.5 S | ≤ 1 S |
| Minocycline | ≤ 0.5 S | ≤ 0.5 S | ≤ 0.5 S |
| Rifampicin | ≤ 0.5 S | ≤ 0.25 S | ≤ 0.5 S |
| Ciprofloxacin | ≤ 1 S | ≤ 0.5 S | ≤ 0.5 S |
| Linezolid | 2 S | 2 S | 2 S |
| Quinupristin/Dalfopristin | ≤ 0.25 S | ≤ 0.25 S | ≤ 0.25 S |
