# Supplementary figures and images for: Heterogeneous vancomycin-intermediate susceptibility in a community-associated methicillin-resistant Staphylococcus aureus epidemic clone, in a case of Infective Endocarditis in Argentina
Source: Ann Clin Microbiol Antimicrob. 2011 Apr 28;10:15. doi: 10.1186/1476-0711-10-15 (PMC3111347; doi:10.1186/1476-0711-10-15)

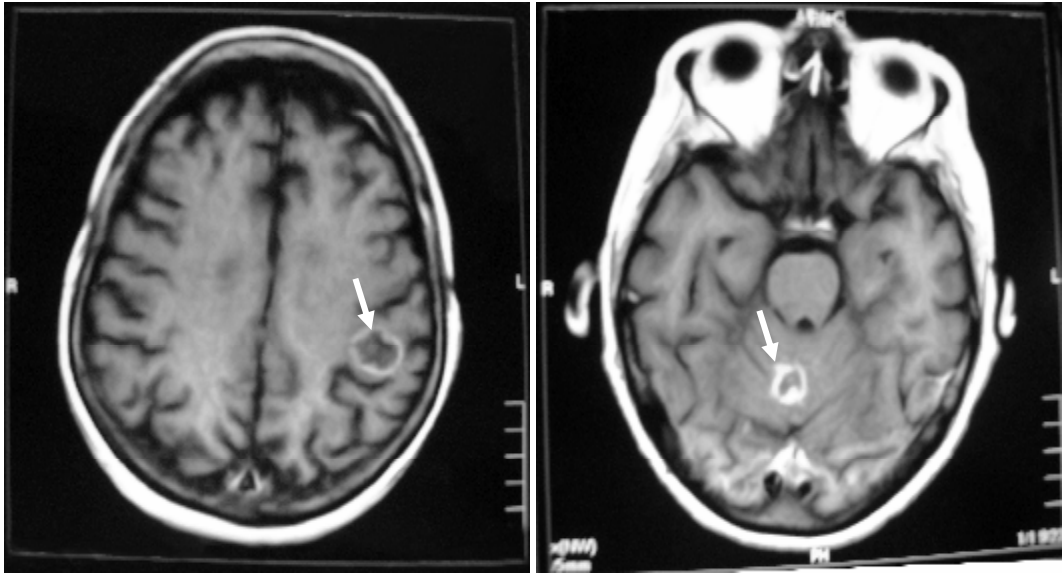

Supplement: Additional file 2 — Figure S1: MRI (two slices) showing brain abscesses (white arrows) in a patient with CA-MRSA infective endocarditis. MRI: magnetic resonance image (with gadolinium). [file 1476-0711-10-15-S2.PDF]

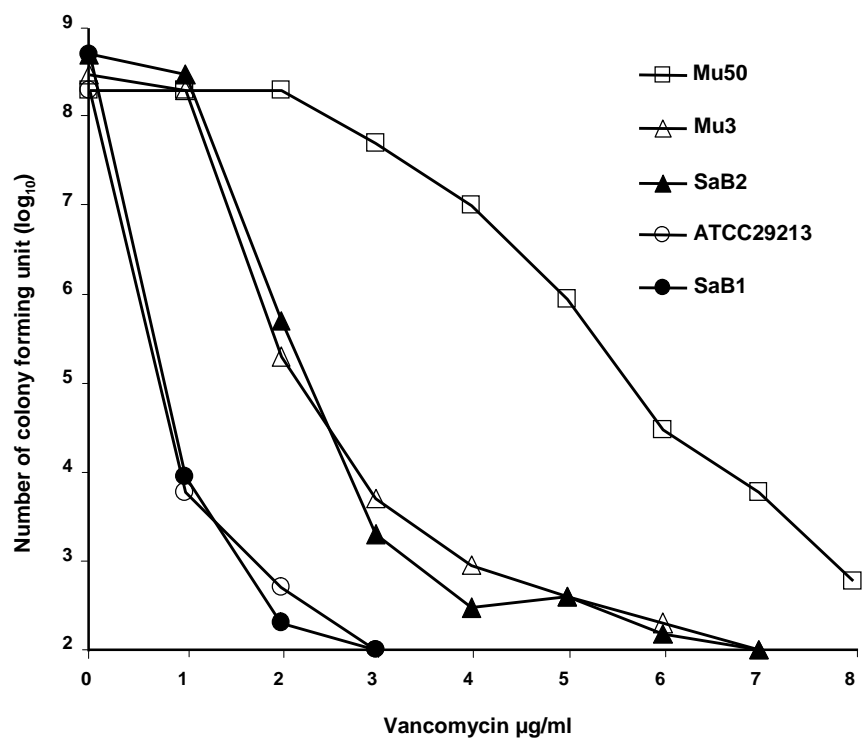

Supplement: Additional file 3 — Figure S2: Vancomycin population analysis profiles of initial isolate-(SaB1)-VSSA and after persistent bacteremia isolate-(SaB2)-h-VISA, despite vancomycin therapy SaB1: initial isolate, prior to vancomycin therapy. Also, hVISA and VISA reference strain Mu3 and Mu50 respectively and VSSA strain ATCC 29213. Briefly, PAP was performed by serial dilution of overnight BHIB culture and inoculation of BHI agar containing 0 to 8 μg/ml of vancomycin. Colonies were counted after incubation for 48 h in air at 35°C and plotted on a graph of the number of CFU/ml versus vancomycin concentration. [file 1476-0711-10-15-S3.PDF]

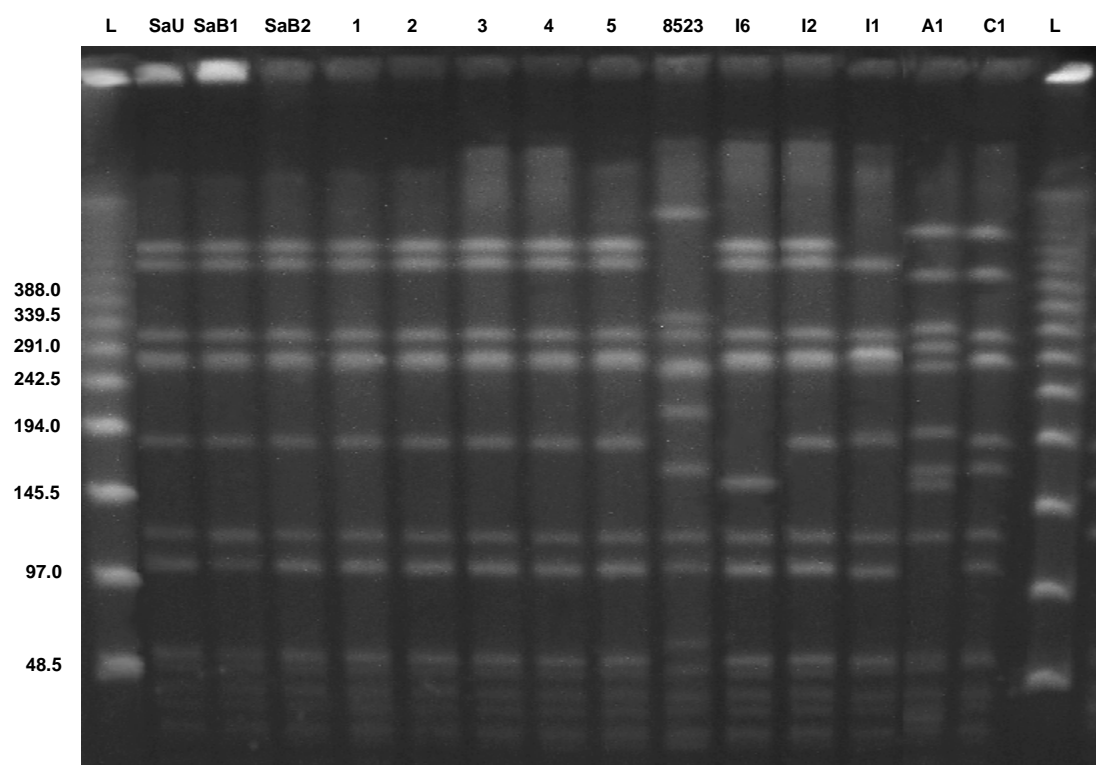

Supplement: Additional file 4 — Figure S3: PFGE-Analysis confirmed the clonality of the clinical isolates (SaU-SaB1-SaB2), belonging to CA-MRSA clone ST5-IV-PVL+. Sma I restriction patterns were indistinguishable for SaU (urine isolate), SaB1 (initial blood isolate) and SaB2 (later blood isolate), 1-5: five derivatives from SaB2 in PAPs (CIM ≥4 μg/ml), PFGE DNA pattern of representative of major clonal types belonging to ST5 lineage, both CA and HA-MRSA from Argentina, to be compared with subtype I2 CA-MRSA (ST5-IV-PVL¯): CA-MRSA I1 (ST5-IV-PVL+), HA-MRSA A1-Cordobes/Chilean clone (ST5-I) and HA-MRSA C1 Pediatric clone (ST100-IV). I6 refers to the PFGE pattern of a MSSA isolate from Córdoba, which shows a unique band difference with the I2 subtype, isolated from our patient. NC: NCTC 8325 control strain L: DNA molecular size markers in kb (lambda DNA ladder, Promega). [file 1476-0711-10-15-S4.PDF]
